# Psychometric Evaluation of the Chinese Version of the Decision Regret Scale

**DOI:** 10.3389/fpsyg.2020.583574

**Published:** 2020-12-03

**Authors:** Richard Huan Xu, Ling Ming Zhou, Eliza Laiyi Wong, Dong Wang, Jing Hui Chang

**Affiliations:** ^1^Centre for Health Systems and Policy Research, Jockey Club School of Public Health and Primary Care, The Chinese University of Hong Kong, Hong Kong, China; ^2^School of Health Management, Southern Medical University, Guangzhou, China

**Keywords:** decisional regret, confirmatory factor analysis, classical test theory, item response theory, China

## Abstract

**Objective:**

The objective of this study was to evaluate the psychometric properties of the Chinese version of the decision regret scale (DRSc).

**Methods:**

The data of 704 patients who completed the DRSc were used for the analyses. We evaluated the construct, convergent/discriminant, and known-group validity; internal consistency and test–retest reliability; and the item invariance of the DRSc. A receiver operating characteristic (ROC) curve was employed to confirm the optimal cutoff point of the scale.

**Results:**

A confirmatory factor analysis (CFA) indicated that a one-factor model fits the data. The internal consistency (α = 0.74) and test–retest reliability [intraclass correlation coefficient (ICC) = 0.71] of the DRSc were acceptable. The DRSc demonstrated unidimensionality and invariance for use across the sexes. It was confirmed that an optimal cutoff point of 25 could discriminate between patients with high and low decisional regret during clinical practice.

**Conclusion:**

The DRSc is a parsimonious instrument that can be used to measure the uncertainty inherent in medical decisions. It can be employed to provide knowledge, offer support, and elicit patient preferences in an attempt to promote shared decision-making.

## Introduction

Effectively engaging patients in medical decision-making is essential for improving their health outcomes, reducing cost and uncertainty, and developing reasonable expectations of the outcome; it can also benefit the clinician’s experience (Brehaut et al., 2003). However, in practice, some medical decisions have to be made on the basis of no clear or clinically preferable options. If the selected decision leads to an unexpected clinical outcome or one that is below expectations, despite the patient’s preferences and needs being respected and considered in the treatment, it is inevitable that patients will experience decisional regret, a very common but negative emotion (Joseph-Williams et al., 2010).

Patients’ decisional regret in the field of healthcare has been only studied during the last few years (Joseph-Williams et al., 2010). The majority have revealed that there is a relationship between medical decisional regret and individual personality, past experience, and the medical professional’s attitude (Tversky and Kahneman, 1981; Zeelenberg et al., 1998; Loewenstein, 2005). For example, Nicolai et al. (2016) found that there is a relationship between the physician’s increased empathy and patient’s reduced decisional regret after treatment. Some other studies discussed the associations between regret and the individual’s quality of life (QoL). For instance, Tanno and Bito (2019) found that, to some extent, the patient’s decision-making depends on his/her perceptions of the QoL after treatment. Clark et al. (2001) also identified a strong relationship between individual regret, treatment choice, and the corresponding QoL. In addition, Feldman-Stewart and Siemens (2015) suggested that decisional regret could be used as a metric to measure the quality of decisions, which could facilitate the performance improvement of the healthcare system. Other studies that have taken a psychological perspective indicated that if regret occurs about a decision, the following “preference reversal” could make the patients favor the other non-chosen option, which might completely counteract their health outcomes (Svenson, 1992; Brehaut et al., 2003). Despite theoretical discussions having resulted in decisional regret being defined in several contexts, the lack of a valid instrument that can be used to measure and quantify medical decisional regret somewhat limits the medical professional’s capability to capture the variations in the patient’s emotions during treatment and to make proper decisions during clinical practice.

Due to the abstract and complex concept of regret, limited instruments have been identified to measure the multiplicity of, and variations in, decisional regret in the field of healthcare; a majority of these have some (or other) methodological concerns, for example, lack of psychometric data, focus only on specific conditions, and assessment of consumer regret rather than patient regret (Joseph-Williams et al., 2010). Among them, the decision regret scale (DRS), which assesses different conceptualizations (e.g., option and outcome regret), is recognized as a valid instrument for measuring the regret of patients who have already made a medical decision (Brehaut et al., 2003). The DRS focuses on patient decisional regret, and it has been translated into several languages and adapted for use in various cultural contexts (Joseph-Williams et al., 2010). However, in China, the measurement of decisional regret in clinical practice is in its infancy (Song et al., 2016). Currently, as few data exist that support studying the influence of regret on patients’ medical decisions, the implementation of patient-centered care (PCC) during clinical practice is negatively affected (Jo Delaney, 2018). Thus, the aim of this study was to evaluate the psychometric properties of the Chinese version of the DRS (DRSc) in order to facilitate the measurement of individual decisional regret in a clinical setting.

## Materials and Methods

### Data Source and Collection

The data used in this study were obtained from a cross-sectional survey that investigated PCC in public hospitals in China from November 2019 to January 2020. Patients were recruited from the inpatient departments of eight hospitals in five cities (Guangzhou, Shenzhen, Zhanjiang, Meizhou, and Shaoguan) in Guangdong Province. All patients from the target hospitals were invited to participate in the study during the appointed survey period. The inclusion criteria for patients were as follows: (1) ≥18 years old, (2) understood Mandarin, (3) had no cognitive problems, and (4) were able to complete the informed consent form. With the assistance of the ward nurses, all of the eligible patients were asked to complete a structured questionnaire during a face-to-face interview, which gathered information about their demographic characteristics, socioeconomic status (SES), health conditions, well-being, use of health services, lifestyle, and attitudes toward PCC. A total of 704 patients who successfully completed the DRSc were used for our psychometric analyses. The study was approved by the institutional review board of the Second Affiliated Hospital of Guangzhou Medical University (ethical approval ID: 2019-ks-28).

### Sample Size

To conduct confirmatory factor analysis (CFA), the minimum sample size that we required was nearly 300 (Floyd and Widaman, 1995; Kline, 2015). For a Rasch analysis, a sample size of 500 is sufficient for analyzing a scale composed of polytomous items (Linacre, 1994). Assuming a Type 1 error of 5% (two−tailed) and a power of 0.80, a total sample size of 704 observations would be able to detect an effect size of *r* = 0.11 in the Pearson product−moment correlation coefficients.

### Instruments

#### Decisional Regret

The DRS is a five-item unidimensional self-reported scale that assesses the patients’ decisional regret (Brehaut et al., 2003). It uses a five-point Likert scale, ranging from 1 to 5, where 1 represents “strongly agree” and 5 “strongly disagree.” The scores of Items 2 and 4 are reversed. The overall score is transformed from 0 to 100 by subtracting 1 from each item and then multiplying by 25. A lower overall score indicates few regrets, whereas a higher overall score indicates more regrets. The original DRS reported a one-factor structure and showed a good internal consistency (Cronbach’s alpha = 0.81–0.92) (Brehaut et al., 2003). The DRSc was directly provided by the research institute of Ottawa Hospital. Ten individuals from the general public were invited for a face-to-face cognitive debriefing to confirm the content and face validity of the DRSc. No further revisions or modifications were needed.

#### Subjective Well-Being

The ICEpop CAPability Measure for Adults (ICECAP-A) is a generic and preference-based instrument that evaluates an individual’s well-being (Al-Janabi et al., 2012). The descriptive system of the ICECAP-A has five items (stability, attachment, autonomy, achievement, and enjoyment), and each item has four response options that range from fully capable to not capable. In this study, we used the item-level score to reflect the patients’ well-being, where a higher score indicated a poor subjective well-being. The psychometric property of the Chinese ICECAP-A was reported by Tang et al.’s (2018) study (Cronbach’s alpha = 0.79). In this study, the Chinese ICECAP-A was provided by the University of Birmingham.

#### Shared Decision-Making (SDM)

The SURE scale (Sure of myself, Understand information, Risk–benefit ratio, and Encouragement) is a four-item questionnaire that screens patients’ decisional conflict during clinical practice (Ferron Parayre et al., 2014). A binary response category is used, with 0 representing “No” and 1 representing “Yes.” The highest overall score of SURE is 4, where less than that indicates the existence of decisional conflict to some extent. The Chinese SURE scale was provided by the research institute of Ottawa Hospital.

The CollaboRATE scale is a three-item questionnaire that measures SDM (Elwyn et al., 2013). The Chinese version of the CollaboRATE contains a scale that ranges from 0 to 10 for each item, where 0 represents “no effort was made” and 10 represents “every effort was made” by the medical professional to promote SDM. The psychometric properties of CollaboRATE have been reported by other studies (Forcino et al., 2018). The Chinese version of CollaboRATE was provided by the developer^1^.

#### Physical and Mental Health Status

The patients’ physical health status was evaluated using a visual analog scale (VAS). A scale ranging between 0 and 100, where 0 represents the worst imaginable health status and 100 represents the best imaginable health status, on the surveying day was presented to them. All of the patients were asked to select the number on the scale that best represented their health status on that day.

Patient Health Questionnaire-2 (PHQ-2) was used to measure the patients’ mental health status. The PHQ-2 includes the first two items of the PHQ-9 (Spitzer et al., 1999), which is the depression module from the full PHQ. The patients were asked to recall the frequency of a depressed mood and anhedonia over the past two weeks. A PHQ-2 score ≥ 3 (score range: 0–6) is recognized as depressive. The psychometric properties of the Chinese version of the PHQ-2 have been reported by other studies (Liu et al., 2016).

### Statistical Analyses

Confirmatory factor analysis was used to investigate the structural validity of the DRSc. The fit of the model was determined using the root-mean-square error of approximation (RMSEA ≤ 0.06), standardized root-mean-square residual (SRMR < 0.08), comparative fit index (CFI > 0.95), and Tucker–Lewis index (TLI > 0.95) (Hu and Bentler, 1999). The Akaike information criterion (AIC) and Bayesian information criterion (BIC) were also employed to compare the performance of the models, with a smaller value indicating a better fit. We formulated *a priori* hypotheses about the relationship between the DRSc and other instruments, such as the ICECAP-A and the SURE, to test both the convergent and divergent validity. Pearson’s correlation coefficient was used to assess the relationships, and the strength of the associations was interpreted as weak (<0.3), moderate (0.3–0.5), and strong (≥0.50) (Cohen, 1992). To examine the known-group validity, the analysis of covariance (ANCOVA) adjusted for sex, age, and the duration of disease (dependent variable was the DRSc overall score) was used to evaluate the between-group differences. We assumed that the patients who were sure about their treatment (overall score of SURE = 4) and showed no depressive disorders (PHQ-2 score < 3) would present low decisional regret and vice versa.

The internal consistency of the DRSc was assessed using Cronbach’s alpha (α > 0.7) and McDonald’s omega, which indicates the strength of the association between items and constructs, as well as the item-specific measurement errors (ω > 0.7) (McDonald, 1999). The item-total correlation (>0.5), average inter-item correlation (0.15–0.5), and alpha if an item was deleted were also reported (DeVellis, 2017). The mean score, standard deviation (*SD*), and ceiling and floor effects of the DRSc scores were calculated. The test–retest reliability was investigated by inviting a minimum of 30 patients to complete the DRSc twice in a 1-week interval period. The intraclass correlation coefficient [ICC (two-way mixed effects model) > 0.7, acceptable] (Fleiss, 1999) and Gwet’s agreement coefficient (Gwet’s AC) were employed to examine the test–retest reliability. Gwet’s AC is used to avoid the “Kappa paradox” (Wongpakaran et al., 2013), which is interpreted as fair (0.21–0.4), moderate (0.41–0.6), good (0.61–0.8), and very good agreement (>0.8) (Landis and Koch, 1977; Wongpakaran et al., 2013).

The partial credit model (PCM), which is a modified Rasch model that can be used with scales that have a polytomous response category, was employed for further analysis. According to the results of CFA, the unidimensional assumption was fulfilled (Pecanac et al., 2018). The Infit and Outfit mean square (MNSQ) statistics, which determine how well each item contributes to defining a single underlying construct, were computed to check whether the items fit the expected model. An MNSQ value ranging between 0.6 and 1.4 indicates adequate item fit (Schumacker and Smith, 2007). The person separation index (PSI > 0.7, acceptable) was calculated to confirm the reliability of the DRSc based on the PCM (Richtering et al., 2017). Differential item functioning (DIF) was employed to check the parameter invariance of the DRSc item performance between the sexes (male vs. female) (Rupp and Zumbo, 2006). It can evaluate the equality of the items and respondent parameters in relation to different populations or measurement conditions (Rupp and Zumbo, 2006). McFadden’s *R*^2^ was used to evaluate the strength of the DIF (<0.13 negligible, 0.13–0.26 moderate, and >0.26 large) (Zumbo, 1999).

A receiver operating characteristic (ROC) analysis was used to determine the optimal cutoff point of the DRSc (Haun et al., 2019). The ROC curve graphically presents the test’s ability to correctly identify the “true-positive” and “true-negative” individuals for various test cutoff points (Haun et al., 2019). We estimated the area under the ROC (AUC) and determined the optimal point based on the Youden index. The R software (R foundation, Vienna, Austria) was used for the data analysis, and the Type I error rate (α) was set at.05 (*p*-value ≤ 0.05).

## Results

### Demographics

In total, 52% of patients were female and the average age was 49.3 years. Regarding the patients’ families, 63.8% were registered as living in urban areas, and 47.0% of patients lived with chronic conditions. Nearly half of the patients reported having a body mass index over 23. Around 65% indicated that the severity of the disease was moderate or higher (Table 1).

**TABLE 1:** Background of respondents (*n* = 704).

	Value
**Sex, *n* (%)**	
Male	337 (48.0)
Female	365 (52.0)
Age (years), mean (*SD*) [range]	49.3 (17.5) [18–91]
**Educational level, *n* (%)**	
No/primary	139 (19.9)
Secondary/post-secondary	427 (61.2)
Tertiary or above	132 (18.9)
**Family register, *n* (%)**	
Urban area	438 (63.8)
Rural area	249 (36.2)
**Living status, *n* (%)**	
Live lone	43 (6.3)
Live with families	644 (93.7)
**Working status, *n* (%)**	
Fully employed	399 (56.8)
Non-employed	89 (12.7)
Retired	214 (21.4)
**Chronic condition, *n* (%)**	
No	323 (47)
Yes	364 (53)
**BMI, *n* (%)**	
≤18.4	68 (9.8)
18.5–22.9	316 (45.5)
≥23	310 (44.7)
**Severity of disease, *n* (%)**	
No threat to life	111 (16.4)
Minor threat to life	130 (19.2)
Moderate threat to life	232 (34.2)
Severe threat to life	205 (30.2)
**Depressive status**	
Yes	297 (46.3)
No	345 (53.7)

*Note: All the respondents who did not complete the personal information but provided the valid responses for the DRSc have been included in the analysis. Two respondents did not provide the information of sex and working status; six did not provide the information of educational level; 17 did not provide the information of family register, living status, and chronic conditions; 10 did not provide the information of BMI; 26 did not provide the information of severity of disease; and 62 did not provide the information of depressive status. SD, standard deviation.*

### Construct Validity

**FIGURE 1:**
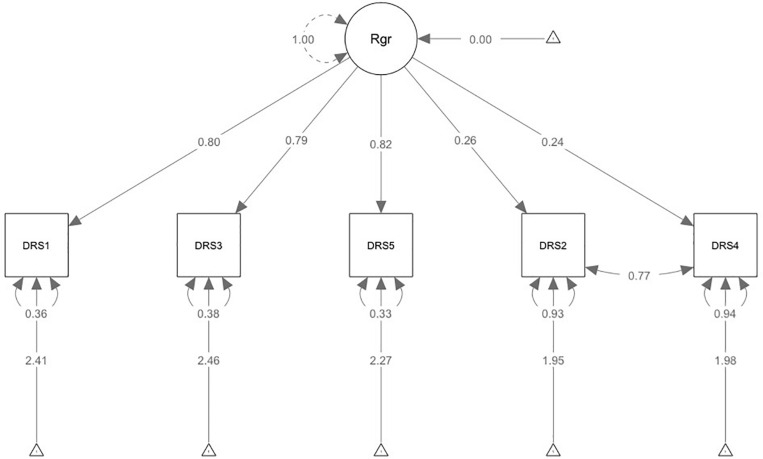
Confirmatory factor analysis model with standardized path coefficients.

Exploratory factor analysis was used to confirm that the model was free of common method bias (the primary factor explained 42% of the total variance). The fit measure of the one-factor model (CFA) indicated some misspecification with χ^2^ (5, *N* = 704) = 628.8, *p* < 0.001, CFI = 0.604, TLI = 0.208, SRMR = 0.181, and RMSEA = 0.421. Residual diagnostics traced this misspecification to the relationship between the indicator residual variances for Items 2 and 4. Hence, we assumed that the non-random measurement error was caused by the reversed wording of these two items, which has been reported in previous studies (Hoyle, 2012). We modified the model and specified the error covariance between Item 2 and Item 4. In the updated model, since χ^2^ (4, *N* = 704) = 11.46, *p* = 0.022, CFI = 0.995, TLI = 0.988, SRMR = 0.01, and RMSEA = 0.051, it performed much better than the first model, and the AIC and BIC also supported this conclusion (Table 2). The second model that included the standardized factor loadings for the observed variables, ranging between 0.24 and 0.82, is presented in Figure 1.

**TABLE 2:** The CFA result of the DRSc.

	Chi-square test			RMSEA	SRMR	CFI	TLI	AIC	BIC
	Value	Degrees of freedom	*p*-value						
One-factor model	628.8	5	<0.001	0.421	0.181	0.604	0.208	8118.6	8164.2
Revised one-factor model	11.46	4	0.022	0.051	0.01	0.995	0.988	7503.3	7553.4

### Item Statistics, Internal Consistency, and Test–Retest Reliability

All of the items showed some floor effects, and these ranged from 27.7% (Item 4) to 47.3% (Item 1). Item 2, which was the “most regrettable” question, had a mean score of 2.34, whereas Item 1, which was the “least regrettable” item, had a mean score of 1.61. The overall mean score was 23.81 (0–100) with an *SD* of 16.25. DRSc showed acceptable internal consistency with α = 0.74 and ω = 0.76. The results of both the ICC (0.71) and Gwet’s AC (0.66–0.81) reflected a good reproducibility of the DRSc (Table 3).

**TABLE 3:** The item statistics and reliability of DRSc.

		Item 1	Item 2	Item 3	Item 4	Item 5	Overall
Response	Strongly agree (floor effect)	47.3	28.55	38.92	27.7	40.2	
	Agree	46.16	34.52	48.72	37.36	45.88	
	Neither agree nor disagree	5.68	18.47	10.942	19.18	12.07	
	Disagree	0.14	11.79	1.14	8.95	0.57	
	Strongly disagree (ceiling effect)	0.71	6.68	0.28	6.82	1.28	
Item statistics	Mean	1.61	2.34	1.75	2.3	1.77	23.81
	Standard deviation	0.67	1.2	0.71	1.16	0.78	16.25
	Skewness	1.25	0.78	0.68	1.15	0.78	0.03
	Kurtosis	3.4	−0.15	−0.46	2.29	0.86	−1.03
Test–retest reliability	ICC (95% confidence interval)						0.71 (0.48–0.85)
	Gwet’s AC	0.81	0.66	0.81	0.77	0.77	
Internal reliability	Alpha if item drop	0.70	0.67	0.70	0.67	0.70	
	Item-total correlation	0.65	0.78	0.65	0.77	0.66	
	Average inter-item correlation	0.42	0.35	0.42	0.34	0.42	
	Cronbach’s alpha						0.74
	McDonald’s omega						0.76

*Note: the score of items 2 and 4 was reversed. Strongly agree means low regret and strongly disagree means high regret. ICC, intraclass correlation coefficient. DRS: Item 1, It was the right decision; Item 2, I regret the choice that was made; Item 3, I would go for the same choice if I had to do it over again; Item 4, The choice did me a lot of harm; and Item 5, The decision was a wise one.*

### Known-Group Validity

As expected, patients who were not sure about their treatment (mean = 31.61) or showed depressive disorders (mean = 28.41) obtained a higher DRSc score than the groups who were sure about their treatment or showed no depressive disorders (Table 4). The results of the ANCOVA indicated that all the differences were statistically significant after being adjusted for sex, age, and the duration of disease (*F*-value = 22.33, *p* < 0.001; *F*-value = 15.47, *p* < 0.001).

**TABLE 4:** The results of known-group validity of the DRSc.

	Mean	*SD*	*F*-value	*p*-value
Sure about decision	21.94	16.15	22.33	<0.001
Not sure about decision	31.61	14.25		
Not depression	23.03	16.28	15.47	<0.001
Depression	28.41	14.50		

*Adjusted by gender, age, and duration. Mean, mean of DRSc; SD, standard deviation.*

### Convergent and Discriminant Validity

The associations between the DRSc and the other measures are shown in Table 5. There was a positive relationship between the DRSc results and the ICECAP-A (0.15–0.18, *p* < 0.001) and PHQ-2 (0.15–0.18, *p* < 0.001), which indicated that more decisional regret resulted in worse well-being or greater depression. The higher DRSc scores were associated with lower SURE (-0.13 to -0.23, *p* < 0.001), CollaboRATE (-0.33, *p* < 0.001), and VAS (-0.17, *p* < 0.001) scores, which suggested that more decisional regret led to higher uncertainty during SDM and a worse physical health status.

**TABLE 5:** The correlation between DRSc and the other measures.

	DRS-Overall
ICECAP-A item 1	0.17***
ICECAP-A item 2	0.18***
ICECAP-A item 3	0.15***
ICECAP-A item 4	0.17***
ICECAP-A item 5	0.16***
SURE1	−0.18***
SURE2	−0.13***
SUER3	−0.19***
SURE4	−0.16***
SURE-Overall	−0.23***
CollaboRATE item 1	−0.33***
CollaboRATE item 2	−0.33***
CollaboRATE item 3	−0.33***
PHQ1	0.15***
PHQ2	0.18***
VAS	−0.17***

*p < 0.05; **p < 0.01; ***p < 0.001.

### PCM and DIF Analyses

Table 6 shows that Infit and Outfit MNSQs of the DRSc ranged between 0.67 and 0.85, which reflected a good fit of the observed data with the model-expected data. The PSI was 0.84, which indicated the good reliability of the DRSc based on the PCM. However, for Items 1, 3, 4, and 5 of the DRSc, the expected category ordering of category 4 and category 5 was not supported by the data, which the last step calibrations did not increase monotonically with category numbers. No item showed DIF across the sex subgroups.

**TABLE 6:** The result of PCM analysis of the DRSc.

	MNSQ Outfit	MNSQ Infit	T1	T2	T3	T4
DRS1	0.67	0.68	−1.05	1.93	3.67	−1.62
DRS2	0.76	0.76	−2.17	0.16	0.36	0.61
DRS3	0.85	0.85	−1.54	1.27	2.24	1.38
DRS4	0.77	0.76	−2.31	0.23	0.69	0.30
DRS5	0.76	0.75	−1.42	1.12	3.03	−0.81
PSI	0.84					

*PSI, person separation index. T1, threshold between category 1 and 2; T2, threshold between category 2 and 3; T3, threshold between category 3 and 4; T4, threshold between category 4 and 5. MNSQ, mean square statistics. DRS: Item 1, It was the right decision; Item 2, I regret the choice that was made; Item 3, I would go for the same choice if I had to do it over again; Item 4, The choice did me a lot of harm; and Item 5, The decision was a wise one.*

### Criterion Validity: ROC Analysis and Cutoff Position Confirmation

**FIGURE 2:**
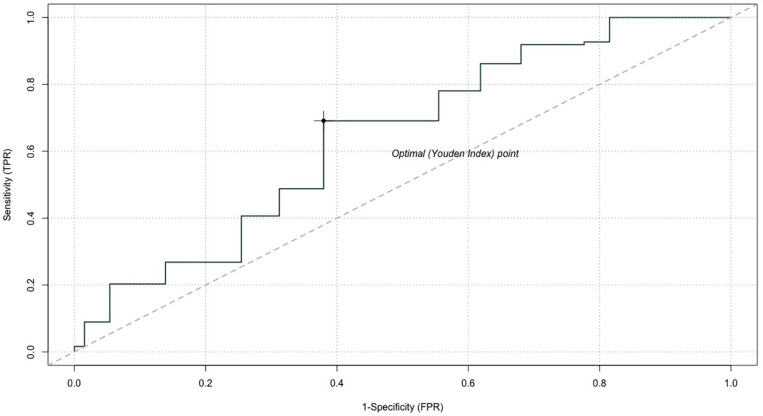
Receiver operating characteristic (ROC) curve for the DRSc. AUC, area under the curve.

The ROC analysis confirmed that the cutoff point of clinically significant decisional regret was based on the patients who had a depressive disorder (as indicated by a PHQ-2 score ≥ 3). The AUC for the DRSc was 64.1%, with a 95% confidence interval of 58.4 and 69.9%, which indicated that the cutoff point was able to accurately discriminate patients with clinically significant regret above random chance (Figure 2). The Youden index, which is a measure of overall diagnostic effectiveness that gives equal weight to sensitivity and specificity, indicated that a score of 25 was linked with a positive predictive value of 0.69 and with a negative predictive value of 0.38 for clinically significant decisional regret. In the survey, 39.8% of patients exceeded this score.

## Discussion

This study evaluated the psychometric properties of the DRSc and confirmed that it is a promising tool for measuring treatment-related decisional regret in China. Overall, the DRSc showed good internal consistency among the patients; it was significantly correlated with other instruments that measure patients’ physical, mental, and social well-being, despite the strength of the correlations not being strong; further, it successfully discriminated between patients who showed different levels of regret about medical decision-making. Therefore, the DRSc could identify a stable construct of regret across a number of different decisions and patients.

The mean score of DRSc was 23.81/100, higher than the average score of 16.5/100 reported in a systematic review of studies that used the DRS (Becerra Pérez et al., 2016). In this study, we used the ROC analysis to determine the clinically meaningful cutoff point of DRSc (25/100). It is the same as the cutoff point between moderate and strong regret as defined by Sheehan et al. (Sheehan et al., 2007), which is accepted by the majority of studies that use DRS. However, given that little clinical evidence exists to support this cutoff point, we suggest interpreting it with caution. Investigations are needed to further confirm the reliability of this cutoff point in different clinical settings and for patients with different medical conditions.

The one-factor structure of DRSc was confirmed, as suggested in the original English version, and this has also been reported by some previous studies, such as the study that assessed the performance of DRS with patients who were receiving an internal cardioverter defibrillator in the United States, and another study that investigated the validity of the Japanese version of DRS (JDRS) (Tanno et al., 2016; Calderon et al., 2019). However, an additional study has indicated that the one-factor structure is unstable because the items of the DRS focus on different concepts; for example, Item 2 appears to target option regret and Item 4 focuses on outcome regret (Joseph-Williams et al., 2010). The measurement of different concepts by the DRS may result in an inconsistency in the explanation and evaluation of regret and this may diminish the power of the measurement. Further explorations are needed. Additionally, although DRSc has an acceptable internal consistency reliability, it is lower than in some other studies that used the DRS. For example, the original DRS study reported that the α value ranges between 0.81 and 0.92 for different patient groups (Brehaut et al., 2003), and the JDRS found an α value of 0.85 (Tanno et al., 2016). However, given that methodologists have suggested that the α value has several limitations when estimating the internal consistency and that it might not be the optimal measure of reliability (Hayes and Coutts, 2020), in this study, we further reported the ω value, which confirmed that the internal consistency of DRSc is acceptable.

While the test–retest reliability of DRS was not considered in the original study, it was tested and confirmed as acceptable in our analysis. In this study, we decided to use a time interval of 1 week, instead of 2 weeks, between the two surveys, which was mainly suggested in previous methodological papers. The first consideration was to avoid the bias created by using different survey methods. In this study, we ensured that the retest survey was conducted in the same way (by the investigator), using the same method (face-to-face interview), and at the same location (ward) as in the first survey, as this could reduce the method effects (DeVellis, 2017). The second consideration in our survey was that more than half of the patients self-reported a poor health status; therefore, a longer time interval may have led to some deterioration in their health and violated the assumption of an unchanged health status that is needed for assessing test–retest reliability, causing inaccuracy in the results (Qian et al., 2020). The previous findings regarding the reproducibility of measuring regret are mixed. Haun et al. (2019) indicated that the reproducibility of DRS was good for caregivers when using an average time interval of 12 weeks. However, another study showed that patients usually change their attitudes toward the original medical decisions (Becerra Pérez et al., 2016). Although regret is an unpleasant emotion, it may result in a positive outcome (Joseph-Williams et al., 2010); for example, it may help to make a better decision in the future. It is necessary to understand the long-term reproducibility of the DRSc to ensure its good capability to measure the consistency of individual decisional regret in different health settings, effectively.

As previous studies indicated, the significant correlations between decisional regret and low QoL and well-being and high depressive disorders were identified in this study (Jokisaari, 2003; Wilson et al., 2016; Xu et al., 2017); however, the strength of correlations was not strong, which indicated a barely satisfactory convergent validity of the DRSc. This finding reflects that regret is a complicated, dynamic psychological process, and a multifaceted concept, which might be strongly affected by the patient’s personality, SES, and health status (Ben-Ezra and Bibi, 2016; Calderon et al., 2019). We found that the patients who reported a high level of regret in decision-making were more likely to have a poor physical and mental health status, which is in line with previous findings. For example, Ratcliff et al. (2013) found that male patients reported greater treatment regret when they had lower sexual and urinary functioning after surgery. Moreover, Becerra-Perez et al. (2016) indicated that a high level of decisional regret was strongly associated with increased decisional conflict. This was also detected in our study as higher DRSc scores were correlated with lower scores in the SDM measures. We further identified a relationship between decisional regret and the patients’ well-being, which has not attracted attention until now. Though a discussion about this relationship was not the aim of this study, it may indicate that evaluation of the treatment outcome should not entirely focus on the physical health gain from curing the disease but also consider the impact of maintaining the patient’s independence, dignity, comfort, and social interaction during their lives. Another reason that the correlations between DRSc and the other measures were not as strong as expected might be that majority of respondents in our study showed a low level of decision regret and the skewness of the DRSc scores might have affected the correlations to some extent.

Overall, the psychometric properties of the DRSc are satisfactory. Nevertheless, there is not without problems. First of all, the performance of Items 2 and 4 needs to be further assessed. Though CFA confirmed a one-factor structure of the DRSc, the lower factor loadings and stronger inter-correlations of these two items than the other three items might imply a two-factor structure of the DRSc. Joseph-Williams et al. (2010) also indicated that Items 2 and 4 focusing on different targets of the regret concept might affect the structure of the DRS. Though limited, the evidence of inconsistencies in measuring decisional regret when using the DRS was also reported by another study (Haun et al., 2019). Additionally, reversed wording might be another reason that affected the construct of the DRS. Considering this was the first study to investigate the performance of the DRS in China, we decided to support the DRSc with one-factor structure and five items with two of them using reversed wording (the results of the two-factor model of the DRSc are presented in the Supplementary Appendix). Further, the results of the Rasch analysis indicated that the order of categories 4 and 5 for four out of five items was inconsistent, which suggested that category 5, i.e., strongly disagree, might not be properly defined for the Chinese population. Remedies such as categorizing the options, collapsing adjacent categories, or removing/revising some items could be considered and evaluated in future studies. Furthermore, despite the DIF analysis showing that the DRSc performed equivalently across the sexes in our sample, a previous study has indicated that males and females tend to show different attitudes toward risk during decision-making (Karakowsky and Elangovan, 2001). Therefore, we suggest that further explorations are required to refine the DRSc using a large sample or for developing a new scale to meet the Chinese population’s preference and needs in measuring the decisional regret in healthcare.

Several limitations should be addressed as well. First, our results might have been affected by some coverage error and potential selection bias due to a non-probability sample being used since all of the patients voluntarily participated in the survey. Second, we did not collect information from non-responding participants, which might have generated selection bias to some extent. Third, all of the information was self-reported, which might have led to some information bias. Fourth, considering the concept of depression is multifaceted, PHQ-2 might not be sensitive enough to measure patients’ mental problems. It might affect the validity of our estimated cutoff point. Fifth, the psychometric properties of the Chinese SURE and CollaboRATE was not assessed, which might affect the convergent validity of the DRSc. Lastly, we did not differentiate between patients with different diseases when they responded to the DRSc, which might have affected the generalizability of our findings.

### Implications

Although the measurement of decisional regret in healthcare has received more attention, this topic has rarely been studied in China. In our study, surveying patients about decisional regret was not easily allowed and was even prohibited by some medical professionals. They were worried that such a conversation would result in a harmful doctor–patient relationship. On the contrary, measuring decisional regret is an important way to understand the patients’ feelings, preferences, expectations, and subsequent decisions when using healthcare services and for achieving PCC.

DRSc is a parsimonious instrument that can measure the uncertainty inherent in medical decisions. It can provide knowledge, offer support, and elicit patient preferences in an attempt to promote SDM. It monitors and assesses the quality of healthcare services based on patient’s perceptions, enhances communication, and facilitates the development of a trusting doctor–patient relationship. Given that talking to patients about decisional regret is sensitive in China, instead of directly asking about their feelings about the treatment decision, the DRSc can provide a prudent way to measure the decisional regret and to understand patients’ real expectations from the treatment outcomes.

## Conclusion

DRSc is proved to be a reliable measurement that has satisfactory validity. It can effectively discriminate between patients who have high and low levels of decisional regret. In this study, a meaningful cutoff point was provided using an ROC analysis in order to facilitate the measurement of decisional regret in both clinical practice and academic studies. DRSc is a psychometrically robust and an easy-to-complete patient-reported outcome measure capable of providing valuable information for supporting PCC in China.

## Data Availability Statement

The raw data supporting the conclusions of this article will be made available by the authors, without undue reservation.

## Ethics Statement

The studies involving human participants were reviewed and approved by the Institutional Review Board of the Second Affiliated Hospital of Guangzhou Medical University (ethical approval ID: 2019-ks-28). The patients/participants provided their written informed consent to participate in this study.

## Author Contributions

RX and LZ contributed to conceptualization, methodology, data collection, writing—original draft, and writing—review and editing. DW, EW, and JC contributed to conceptualization, writing—review and editing, and supervision. All authors contributed to the article and approved the submitted version.

## Conflict of Interest

The authors declare that the research was conducted in the absence of any commercial or financial relationships that could be construed as a potential conflict of interest.

## Footnotes

1 http://www.glynelwyn.com
