# Supplementary material for: Psychometric Evaluation of the Chinese Version of the Decision Regret Scale
Source: Front Psychol. 2020 Dec 3;11:583574. doi: 10.3389/fpsyg.2020.583574 (PMC7793926; doi:10.3389/fpsyg.2020.583574)
Supplement: Supplementary file 1 [file Data_Sheet_1.pdf]

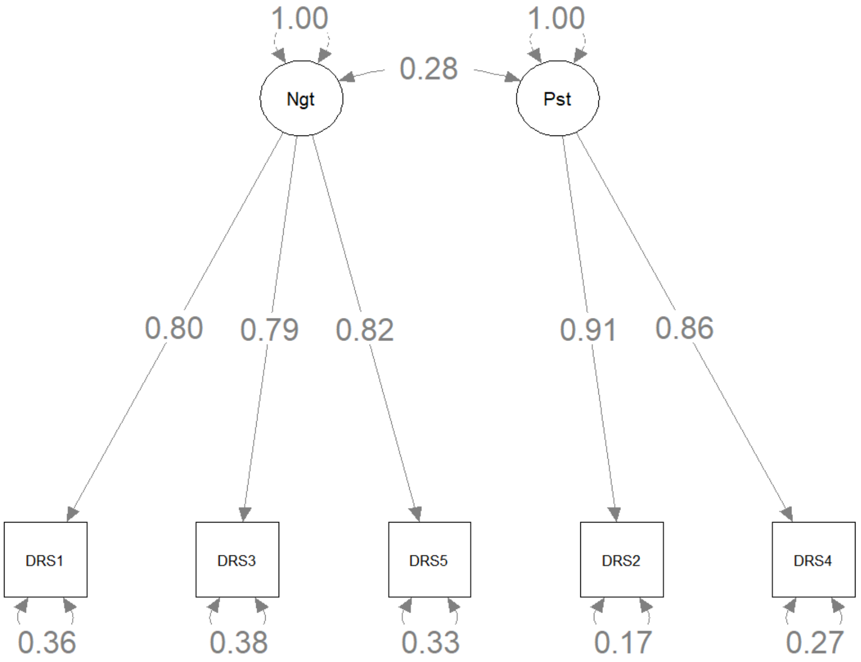

Figure A-1 Two-factor model of the DRSc; Items 1, 3, and 5 were summarized as the dimension of negative wording; Items 2 and 4 were summarized as the dimension of positive wording

Table A-1 The result of CFA of 2-factor model

	Chi-square test			RMSEA	SRMR	CFI	TLI	AIC	BIC
	Value	Degrees of freedom	*p*-value						
Two-factor model	11.457	4	0.022	0.051	0.01	0.995	0.988	7503.259	7553.384
